# Effect of High *N*-Acetylcysteine Concentrations on Antibiotic Activity against a Large Collection of Respiratory Pathogens

**DOI:** 10.1128/AAC.01334-16

**Published:** 2016-11-21

**Authors:** Giulia Landini, Tiziana Di Maggio, Francesco Sergio, Jean-Denis Docquier, Gian Maria Rossolini, Lucia Pallecchi

**Affiliations:** a Department of Medical Biotechnologies, University of Siena, Siena, Italy; b Corporate Respiratory Medical Affairs, Zambon S.p.A., Bresso, Italy; c Department of Experimental and Clinical Medicine, University of Florence, Florence, Italy; d Clinical Microbiology and Virology Unit, Florence Careggi University Hospital, Florence, Italy; e Don Carlo Gnocchi Foundation, Florence, Italy

## Abstract

The effect of high *N*-acetylcysteine (NAC) concentrations (10 and 50 mM) on antibiotic activity against 40 strains of respiratory pathogens was investigated. NAC compromised the activity of carbapenems (of mostly imipenem and, to lesser extents, meropenem and ertapenem) in a dose-dependent fashion. We demonstrated chemical instability of carbapenems in the presence of NAC. With other antibiotics, 10 mM NAC had no major effects, while 50 mM NAC sporadically decreased (ceftriaxone and aminoglycosides) or increased (penicillins) antibiotic activity.

## TEXT

*N*-Acetylcysteine (NAC) is a mucolytic agent with antioxidant and anti-inflammatory properties (1–3). Accumulating evidence also points to intrinsic antimicrobial and antibiofilm activities in some cases (4). Due to its properties, NAC is commonly administered together with antibiotics for the treatment of lower respiratory tract infections, and there is a growing interest in also evaluating its roles in the management of cystic fibrosis (CF) and other chronic respiratory diseases (2, 5, 6).

In this perspective, it is crucial to elucidate any potential modulatory effect of NAC on antibiotic activity, which has been a matter of debate (7–12). In particular, discordant results were recently reported from two studies that investigated the effect of 10 mM NAC (i.e., 1.6 mg/ml) on the activity of some antibiotics against a few Gram-negative pathogens (including Escherichia coli, Klebsiella spp., Pseudomonas aeruginosa, and Acinetobacter baumannii). Indeed, the modulatory effects observed by Goswami et al. (10) (i.e., synergism with ampicillin and antagonism with fluoroquinolones, aminoglycosides, and macrolides) were not confirmed by Rodríguez-Beltrán et al. (11), who demonstrated that the modulation of the activity of fluoroquinolones and aminoglycosides was actually related to the low pH of pure NAC powder solutions and not to NAC itself, suggesting that pH-related issues likely contributed to the inconsistency of data from previous studies. In their work, Rodríguez-Beltrán et al. (11) found that only the activity of imipenem was significantly affected by NAC, and they proposed that, for P. aeruginosa, this antagonism was due to competitive inhibition of imipenem uptake through outer membrane porin D (OprD) by NAC.

Considering the relevance of a potential antagonistic effect of NAC on antibiotic activity, especially with long-term high-dosage topical administration (as should be the case for chronic respiratory diseases [4]), we performed a study to evaluate the effect of high NAC concentrations on the activity of several antibiotics against a large collection of respiratory pathogens. In this study, we used 10 and 50 mM NAC concentrations, which represented the concentration tested in previous studies and a 5-fold higher concentration, respectively. The latter concentration was used since it was suggested that concentrations of active NAC as high as 29 mM can be achieved after a single topical administration of 10% NAC (13), and even higher concentrations could be achieved by multiple-dosing regimens, by using 20% NAC for nebulization, or by following direct instillation.

A total of 40 reference and clinical strains of the bacterial species primarily responsible for respiratory tract infections were analyzed (Table 1). MICs of the most relevant therapeutic options for the different pathogens (Table 2; see also Tables S1 to S6 in the supplemental material) were determined in the absence or presence of 10 and 50 mM NAC using the reference broth microdilution method (14). All experiments were carried out at least in duplicate. NAC stock solutions (100 mg/ml) were prepared immediately before their use by dissolving air-protected samples of NAC powder (Zambon S.p.A., Bresso, Italy) in sterile double-distilled water, adjusting the pH to 6.5 with NaOH, and filtering through a 0.22-μm membrane filter. The stability of 200 μM imipenem, meropenem, and ertapenem solutions in the absence or presence of NAC was investigated in phosphate-buffered saline (PBS) (pH 7.4) at 25°C and 37°C and in cation-adjusted Mueller-Hinton broth (CAMHB) at 37°C. For this purpose, absorbance at 300 nm was recorded for up to 6 h using an Envision microplate reader (Perkin-Elmer, Waltham, MA, USA). l-Cysteine, a thiol compound known to promote carbapenem hydrolysis (14, 15), was used as a comparator in experiments performed in PBS at 25°C and tested at the same molar concentrations. The effect of NAC on the bactericidal activity of carbapenems was investigated by time-kill assays (16) using E. coli ATCC 25922 as a test strain and imipenem at a concentration of 8 μg/ml.

**TABLE 1:** Origin and main features of the 40 strains included in the study

Strain	Origin and main features^*a*^
E. coli ATCC 25922	ATCC reference strain
E. coli Z21	Cystic fibrosis (2-yr lung colonization), ESBL-positive ST131 clinical isolate
E. coli Z24	Cystic fibrosis clinical isolate
E. coli Z25	Lower respiratory tract infection, ESBL-positive KPC-positive clinical isolate
K. pneumoniae ATCC 700603	ATCC ESBL-positive reference strain
K. pneumoniae NTUH-K2044	Liver abscess and meningitis, capsular serotype K1, hypermucoviscous (see reference 17)
K. pneumoniae CIP 52.145	CIP reference strain, capsular serotype K2, hypermucoviscous
K. pneumoniae Z4	Cystic fibrosis (1-yr lung colonization), ESBL-positive AmpC-positive clinical isolate
K. pneumoniae Z11	Lower respiratory tract infection, KPC-positive clinical isolate
Klebsiella oxytoca CCUG 15717^T^	CCUG type strain
Enterobacter cloacae CIP 6085^T^	CIP type strain
E. cloacae Z16	Cystic fibrosis ESBL-positive clinical isolate
E. cloacae Z17	Lower respiratory tract infection clinical isolate
E. cloacae Z18	Lower respiratory tract infection clinical isolate
E. cloacae Z19	Lower respiratory tract infection clinical isolate
P. aeruginosa ATCC 27853	ATCC reference strain
P. aeruginosa PAO-1	P. aeruginosa reference strain (see reference 18)
P. aeruginosa Z32	Lower respiratory tract infection clinical isolate
P. aeruginosa Z34	Cystic fibrosis (3-yr lung colonization) clinical isolate
P. aeruginosa Z38	Acute bacterial rhinosinusitis clinical isolate
A. baumannii ATCC 17978	ATCC reference strain
A. baumannii RUH 134	Reference strain for the global clone 2 (see reference 19)
Moraxella catarrhalis Z72	Lower respiratory tract infection clinical isolate
M. catarrhalis Z73	Lower respiratory tract infection clinical isolate
H. influenzae ATCC 49247	ATCC reference strain
H. influenzae Z83	Lower respiratory tract infection clinical isolate
Staphylococcus aureus ATCC 25923	ATCC reference MSSA strain
S. aureus ATCC 6538	ATCC reference MSSA strain
S. aureus ATCC 43300	ATCC reference MRSA strain
S. aureus MRSA-IT1	Bloodstream infection, MRSA, hVISA (see reference 20)
S. aureus Z57	Acute bacterial rhinosinusitis, MSSA clinical isolate
S. aureus Z61	Lower respiratory tract infection, MSSA clinical isolate
Streptococcus pyogenes ATCC 12344^T^	ATCC type strain
S. pyogenes Z90	Lower respiratory tract infection clinical isolate
S. pyogenes Z91	Cellulitis clinical isolate
Streptococcus pneumoniae ATCC 49619	ATCC reference strain
S. pneumoniae Z104	Lower respiratory tract infection clinical isolate
S. pneumoniae Z105	Lower respiratory tract infection clinical isolate
C. striatum Z114	Lower respiratory tract infection clinical isolate
C. striatum Z115	Lower respiratory tract infection clinical isolate

a When available, the main features concerning resistance determinants and molecular typing were reported. ATCC, American Type Culture Collection; CIP, Collection of Institut Pasteur; CCUG, Culture Collection, University of Goteborg; ESBL, extended-spectrum β-lactamase; KPC, Klebsiella pneumoniae carbapenemase; AmpC, AmpC-like β-lactamase; MSSA, methicillin-susceptible S. aureus; MRSA, methicillin-resistant S. aureus; hVISA, heterogeneous vancomycin-intermediate S. aureus.

**TABLE 2:**
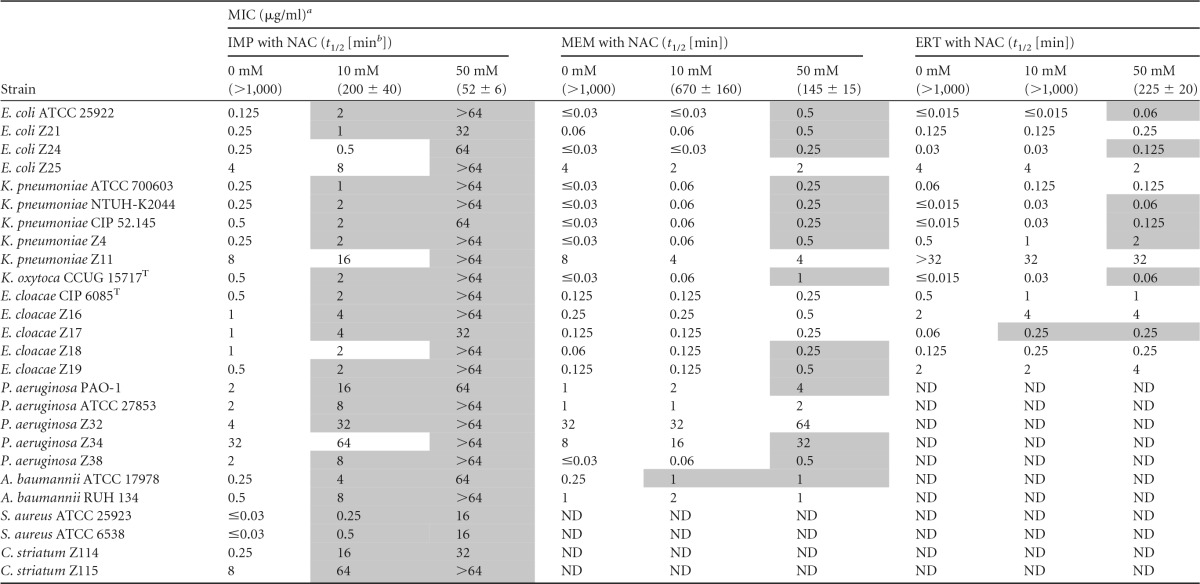
MICs of carbapenems in the absence and those in the presence of NAC for a panel of respiratory pathogens

^a^ MIC changes of >2-fold dilution in the presence of NAC are shaded. IMP, imipenem; MEM, meropenem; ERT, ertapenem; ND, not determined because the species is intrinsically resistant, breakpoints are lacking, or the drug is not a preferred option for that species.

^b^ Half-life values (*t*_1/2_) of carbapenem solutions in CAMHB (37°C) in the absence or presence of NAC are reported.

**FIG 1:**
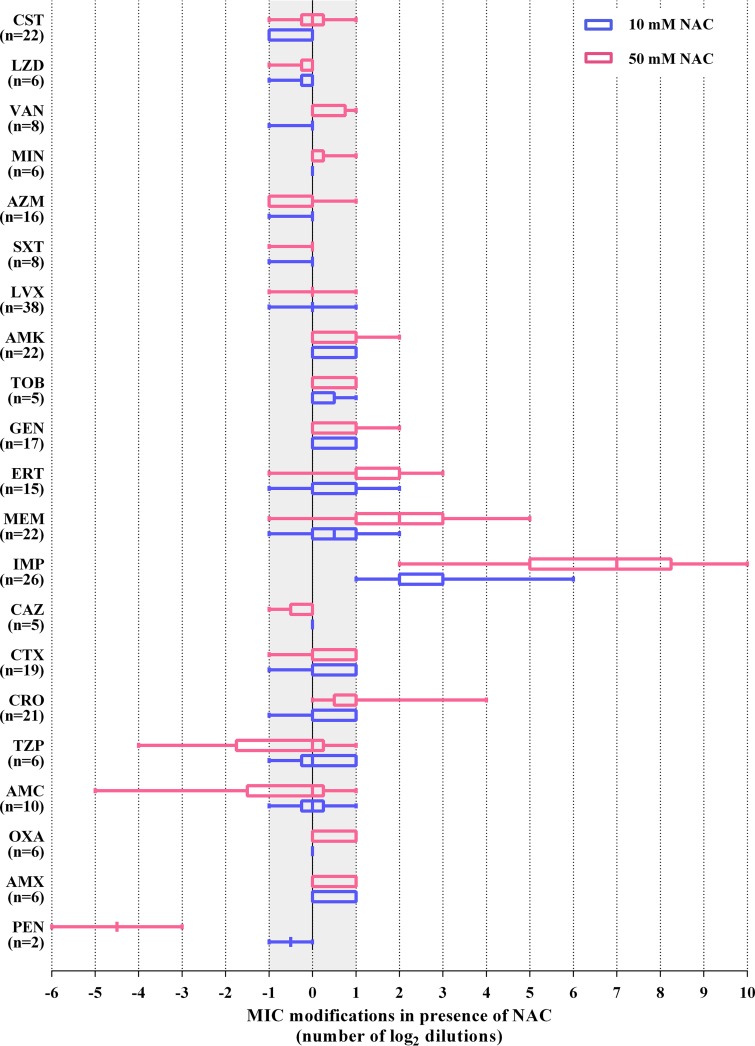
Effects of two high NAC concentrations (10 mM and 50 mM) on antibiotic activity against a collection of respiratory pathogens. A modulatory effect on antibiotic activity was defined as an MIC modification of at least 2-log_2_ dilutions. The number of strains tested for each antibiotic is indicated in parentheses, while details on bacterial species and MIC results are reported in Table 1, Table 2, and supplemental Tables S1 to S6. To express the results as log_2_ dilution variations, a value corresponding to the lowest or to twice that of the highest antibiotic concentration tested was assigned to MICs that could not be determined because out-of-range antibiotic concentrations were used. This is particularly relevant for MICs of imipenem in the presence of 50 mM NAC, which was >64 μg/ml for the majority of isolates tested. PEN, penicillin; AMX, amoxicillin; OXA, oxacillin; AMC, amoxicillin-clavulanic acid; TZP, piperacillin-tazobactam; CRO, ceftriaxone; CTX, cefotaxime; CAZ, ceftazidime; IMP, imipenem; MEM, meropenem; ERT, ertapenem; GEN, gentamicin; AMK, amikacin; TOB, tobramycin; LVX, levofloxacin; SXT, trimethoprim-sulfamethoxazole; AZM, azithromycin; MIN, minocycline; VAN, vancomycin; LZD, linezolid; CST, colistin. The box-and-whisker plot (with boxes extending from the 25th to 75th percentiles and whiskers indicating the minimum and maximum values) was generated by GraphPad Prism 5 (GraphPad, La Jolla, CA).

**FIG 2:**
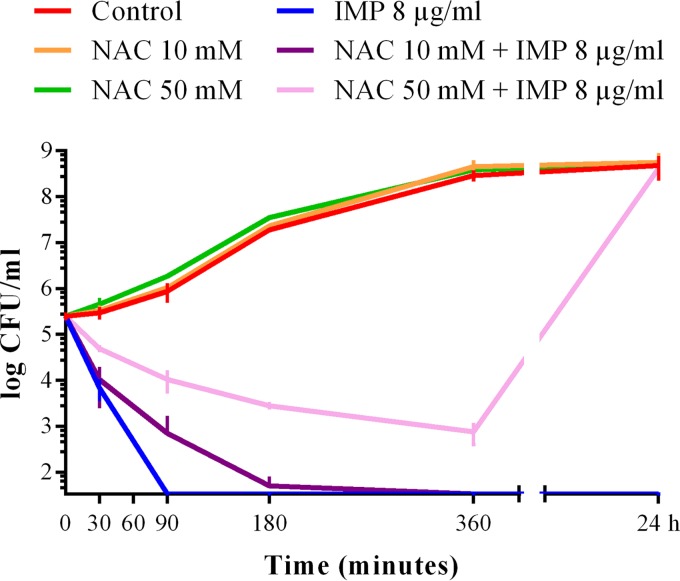
Time-kill curves of imipenem (8 μg/ml) alone and in combination with 10 and 50 mM NAC against E. coli ATCC 25922. The *x* axis is set at the experimental detection limit (1.5-log CFU/ml).

The MIC of NAC was >16 mg/ml for each tested strain except for the two Haemophilus influenzae strains, each of which had an MIC of 16 mg/ml. In the presence of either 10 or 50 mM NAC, the MICs of most antibiotics remained within a single log_2_ dilution difference (i.e., the commonly accepted range of experimental reproducibility), with the notable exception of carbapenems (Fig. 1 and Table 2; see also Tables S1 to S6). Indeed, a clear dose-dependent inhibition of carbapenem activity by NAC was observed with all tested isolates, with imipenem affected more than meropenem and ertapenem, for which the activity was preserved with 10 mM NAC (Fig. 1 and Table 2). At the higher concentration (50 mM), NAC occasionally increased the MICs of ceftriaxone, gentamicin, and amikacin for some enterobacterial strains (Fig. 1; see also Table S1). In contrast, 50 mM NAC decreased the MICs of penicillin and amoxicillin-clavulanic acid for both Corynebacterium striatum strains and the MIC of piperacillin-tazobactam for one Klebsiella pneumoniae strain (Fig. 1; see also Table S1 and S6). Time-kill assays confirmed a dose-dependent inhibition by NAC of the bactericidal activity of imipenem against E. coli ATCC 25922 (Fig. 2).

Although carbapenems were significantly more stable in the presence of NAC (in which the nucleophilicity of the thiol group is reduced by the addition of an *N*-acetyl substituent) than in the presence of l-cysteine (see Table S7), their stability in PBS or in CAMHB was consistently affected in the presence of 10 or 50 mM NAC (Table 2; see also Table S7). Notably, imipenem was less stable than meropenem or ertapenem in each tested condition (Table 2; see also Table S7). Temperature and medium impacted NAC-mediated carbapenem inactivation, with a more rapid inactivation observed at 37°C than at 25°C and in CAMHB than in PBS (see Table S7). Overall, these data are consistent with the impact of NAC on carbapenem MIC values (Fig. 1 and Table 2). Nonetheless, some heterogeneity in the antagonistic effect of NAC for carbapenems among the tested strains was observed, suggesting that additional mechanisms might contribute to the observed NAC-mediated increase in carbapenem MICs, including issues related to carbapenem permeability, as previously suggested for P. aeruginosa (11).

In conclusion, the results of this study demonstrate that high NAC concentrations (possibly reached in the airways following topical administration) overall do not interfere with the activity of the most commonly used antibiotics, with the exception of carbapenems. The instability of imipenem and, to a lesser extent, of meropenem and ertapenem in the presence of high NAC concentrations was a major mechanism accounting for the antagonistic interaction with carbapenems. However, considering that the MICs of meropenem and ertapenem for susceptible strains remained mostly below the susceptibility breakpoints even in the presence of 50 mM NAC, the negative interaction of NAC with these drugs might not be clinically relevant and warrants further evaluation. A modulatory effect on the activity of other beta-lactams and aminoglycosides was also observed at the highest NAC concentration tested with some strains, resulting in an either synergistic or antagonistic interaction. Interestingly, NAC showed intrinsic antimicrobial activity against H. influenzae, which deserves further investigation.

## Supplementary Material

Supplemental material
