# Supplementary material for: Effect of High N-Acetylcysteine Concentrations on Antibiotic Activity against a Large Collection of Respiratory Pathogens
Source: Antimicrob Agents Chemother. 2016 Nov 21;60(12):7513–7. doi: 10.1128/AAC.01334-16 (PMC5119039; doi:10.1128/AAC.01334-16)
Supplement: Supplemental material [file supp_60_12_7513__index.html]

Supplemental material 

# Effect of High *N*-Acetylcysteine Concentrations on Antibiotic Activity against a Large Collection of Respiratory Pathogens

## Supplemental material

**Files in this Data Supplement:**

- Supplemental file 1 -

  Supplemental Tables S1 to S7

  PDF, 787K
