# Supplementary material for: Effect of High N-Acetylcysteine Concentrations on Antibiotic Activity against a Large Collection of Respiratory Pathogens
Source: Antimicrob Agents Chemother. 2016 Nov 21;60(12):7513–7. doi: 10.1128/AAC.01334-16 (PMC5119039; doi:10.1128/AAC.01334-16)
Supplement: Supplemental material [file AAC.01334-16_zac012165770so1.pdf]

SUPPLEMENTAL TABLE S1 MICs (μg/ml) of antibiotics other than carbapenems, determined in absence and presence of two high NAC concentrations: *Enterobacteriaceae*

Antibiotic	AMC			TZP			CTX			CRO			GEN			AMK			LVX			CST		
NAC (mM)	0	10	50	0	10	50	0	10	50	0	10	50	0	10	50	0	10	50	0	10	50	0	10	50
E. coli ATCC 25922	8	16	16	nd	nd	nd	0.06	0.125	0.06	0.06	0.06	0.25	1	1	1	1	2	2	≤0.03	≤0.03	≤0.03	0.5	0.5	0.5
E. coli Z21	64	64	64	nd	nd	nd	>512	>512	>512	>512	>512	>512	2	4	4	16	32	32	64	64	32	1	0.5	0.5
E. coli Z24	8	8	8	nd	nd	nd	0.25	0.125	0.25	0.125	0.125	1	2	2	2	8	16	16	64	64	64	0.5	0.5	0.5
E. coli Z25	>64	64	>64	nd	nd	nd	>512	>512	>512	>512	>512	>512	>256	>256	>256	32	32	64	16	16	16	1	0.5	0.5
Kp ATCC 700603	nd	nd	nd	16	16	8	4	8	4	4	8	8	8	16	16	1	2	2	1	0.5	1	1	1	1
Kp NTUH-K2044	nd	nd	nd	2	4	2	0.06	0.06	0.125	0.06	0.06	0.125	0.25	0.5	1	2	2	4	0.06	0.125	0.06	0.5	0.5	1
Kp CIP 52.145	nd	nd	nd	2	2	2	0.06	0.06	0.06	0.06	0.06	0.25	0.25	0.5	1	1	2	4	0.06	0.06	0.06	0.5	0.5	1
Kp Z4	nd	nd	nd	64	128	128	512	>512	512	>512	>512	>512	>256	>256	>256	>64	>64	>64	1	1	1	0.5	0.5	1
Kp Z11	nd	nd	nd	>256	256	32	64	64	128	64	64	128	4	8	8	32	64	64	64	32	32	1	0.5	1
K. oxytoca CCUG 15717^{T}	nd	nd	nd	4	4	4	0.06	0.06	0.06	0.125	0.25	0.25	1	2	1	2	4	4	0.06	0.06	0.06	0.5	0.5	0.5
E. cloacae CIP 6085^{T}	nd	nd	nd	nd	nd	nd	8	8	4	8	4	16	0.5	1	1	1	2	2	≤0.03	≤0.03	≤0.03	64	64	64
E. cloacae Z16	nd	nd	nd	nd	nd	nd	256	512	512	512	>512	>512	>256	>256	>256	>64	>64	>64	0.5	0.5	0.5	1	0.5	2
E. cloacae Z17	nd	nd	nd	nd	nd	nd	0.06	0.125	0.125	0.06	0.125	1	0.5	0.5	1	0.5	1	1	≤0.03	≤0.03	≤0.03	64	64	32
E. cloacae Z18	nd	nd	nd	nd	nd	nd	0.25	0.25	0.125	0.5	0.5	1	0.5	1	2	2	2	4	0.06	0.06	0.06	0.5	0.5	0.5
E. cloacae Z19	nd	nd	nd	nd	nd	nd	256	256	128	512	>512	>512	256	>256	>256	1	2	2	32	32	32	0.5	0.5	1

Kp, *Klebsiella pneumoniae*; AMC, amoxicillin-clavulanic acid; TZP, piperacillin-tazobactam; CTX, cefotaxime; CRO, ceftriaxone; GEN, gentamicin; AMK, amikacin; LVX, levofloxacin; CST, colistin; nd, not determined. MIC changes by more than one 2-fold dilution in the presence of NAC are shaded.

SUPPLEMENTAL TABLE S2 MICs (µg/ml) of antibiotics other than carbapenems, determined in absence and presence of two high NAC concentrations: *Pseudomonas aeruginosa* and *Acinetobacter baumannii*

Antibiotic	CAZ			TOB			AMK			LVX			CST		
NAC (mM)	0	10	50	0	10	50	0	10	50	0	10	50	0	10	50
P. aeruginosa PAO-1	0.5	0.5	0.5	0.25	0.5	0.5	1	2	2	1	1	1	2	2	2
P. aeruginosa ATCC 27853	1	1	1	0.5	0.5	0.5	2	2	2	1	1	1	2	1	1
P. aeruginosa Z32	8	8	8	0.5	0.5	0.5	1	1	1	2	1	1	0.5	0.5	0.5
P. aeruginosa Z34	128	128	64	32	32	32	16	16	16	8	8	4	2	1	1
P. aeruginosa Z38	1	1	1	0.5	0.5	1	2	4	4	0.125	0.125	0.125	1	1	1
A. baumannii ATCC 17978	nd	nd	nd	nd	nd	nd	2	2	2	0.25	0.25	0.125	1	1	1
A. baumannii RUH 134	nd	nd	nd	nd	nd	nd	4	4	4	0.25	0.25	0.25	1	1	1

CAZ, ceftazidime; TOB, tobramycin; AMK, amikacin; LVX, levofloxacin; CST, colistin; nd, not determined.

Supplemental Table S3 MICs (µg/ml) of antibiotics determined in absence and presence of two high NAC concentrations: *Moraxella catarrhalis* and *Haemophilus influenzae*

Antibiotic	AMC			CTX			LVX			AZM			SXT		
NAC (mM)	0	10	50	0	10	50	0	10	50	0	10	50	0	10	50
M. catarrhalis Z72	0.5	0.5	1	0.5	0.5	0.5	0.06	0.06	0.06	0.06	0.06	0.03	nd	nd	nd
M. catarrhalis Z73	0.25	0.25	0.125	0.25	0.25	0.5	0.06	0.06	0.06	0.06	0.03	0.03	nd	nd	nd
H. influenzae ATCC 49247	8	8	8	0.5	0.5	1	0.03	0.03	0.03	nd	nd	nd	0.125	0.125	0.125
H. influenzae Z83	1	2	1	0.125	0.125	0.25	0.015	0.03	0.03	nd	nd	nd	>16	>16	>16

AMC, amoxicillin-clavulanic acid; CTX, cefotaxime; LVX, levofloxacin; AZM, azithromycin; SXT, trimethoprim-sulfamethoxazole; nd, not determined.

Supplemental Table S4 MICs (µg/ml) of antibiotics other than carbapenems, determined in absence and presence of two high NAC concentrations:

Staphylococcus aureus

Antibiotic	OXA			LVX			SXT			AZM			MIN			VAN			LZD		
NAC (mM)	0	10	50	0	10	50	0	10	50	0	10	50	0	10	50	0	10	50	0	10	50
S. aureus ATCC 6538	0.125	0.125	0.25	0.125	0.25	0.125	0.125	0.125	0.125	1	1	0.5	0.06	0.06	0.06	1	1	2	2	2	2
S. aureus ATCC 25923	0.25	0.25	0.25	0.25	0.25	0.25	0.125	0.06	0.06	1	1	0.5	0.125	0.125	0.125	2	2	2	2	2	2
S. aureus ATCC 43300	16	16	32	0.25	0.25	0.25	0.125	0.125	0.125	>256	>256	>256	0.06	0.06	0.06	1	1	2	2	2	2
S. aureus MRSA-IT1	8	8	8	8	8	8	0.125	0.125	0.125	2	2	1	0.06	0.06	0.06	2	2	2	4	2	2
S. aureus Z57	0.5	0.5	0.5	0.125	0.125	0.125	0.125	0.125	0.125	1	1	1	0.06	0.06	0.125	1	1	1	4	4	4
S. aureus Z61	0.5	0.5	0.5	0.125	0.125	0.125	0.125	0.125	0.125	2	2	2	0.125	0.125	0.125	2	1	2	4	4	4

OXA, oxacillin; LVX, levofloxacin; SXT, trimethoprim-sulfamethoxazole; AZM, azithromycin; MIN, minocycline; VAN, vancomycin; LZD, linezolid.

SUPPLEMENTAL TABLE S5 MICs (µg/ml) of antibiotics determined in absence and presence of two high NAC concentrations: *Streptococcus pyogenes* and *Streptococcus pneumoniae*

Antibiotic	AMX			CRO			LVX			AZM		
NAC (mM)	0	10	50	0	10	50	0	10	50	0	10	50
S. pyogenes ATCC 12344[T]	0.03	0.03	0.03	0.03	0.03	0.03	0.5	0.5	0.5	0.125	0.125	0.125
S. pyogenes Z90	≤0.008	0.015	0.015	0.015	0.03	0.03	0.5	0.5	0.5	0.125	0.125	0.125
S. pyogenes Z91	0.015	0.03	0.015	0.015	0.03	0.03	0.5	0.5	0.5	0.25	0.125	0.25
S. pneumoniae ATCC 49619	0.06	0.125	0.125	0.125	0.125	0.125	0.5	0.5	0.5	0.06	0.06	0.125
S. pneumoniae Z104	0.015	0.03	0.03	0.03	0.06	0.06	1	1	1	32	32	32
S. pneumoniae Z105	≤0.008	≤0.008	0.015	0.06	0.06	0.125	1	1	1	32	32	32

AMX, amoxicillin; CRO, ceftriaxone; LVX, levofloxacin; AZM, azithromycin. MICs values were always confirmed by CFU count, required due to the alteration of culture medium (containing 5% lysed horse blood) color in the presence of high NAC concentrations.

Supplemental Table S6 MICs (µg/ml) of antibiotics other than carbapenems, determined in absence and presence of two high NAC concentrations: *Corynebacterium striatum*

Antibiotic	PEN			AMC			GEN			AZM			VAN		
NAC (mM)	0	10	50	0	10	50	0	10	50	0	10	50	0	10	50
C. striatum Z114	2	2	0.25	2	2	0.25	8	8	16	>32	>32	>32	0.5	0.5	0.5
C. striatum Z115	32	16	0.5	32	16	1	4	4	8	>32	>32	>32	0.5	0.5	0.5

PEN, penicillin; AMC, amoxicillin-clavulanic acid; GEN, gentamicin; AZM, azithromycin; VAN, vancomycin. MIC changes by more than one 2-fold dilution in the presence of NAC are shaded. MICs values were always confirmed by CFU count, required due to the alteration of culture medium (containing 5% lysed horse blood) color in the presence of high NAC concentrations.

SUPPLEMENTAL TABLE S7 Stability of carbapenems in the presence of NAC.

Compound (experimental conditions)	Antibiotic $t_{1/2}$ (min)		
	Imipenem	Meropenem	Ertapenem
None (PBS, 25°C)	>1000	>1000	>1000
10 mM L-cysteine (PBS, 25°C)	9.5 ± 0.1	10.4 ± 0.1	21.3 ± 0.5
50 mM L-cysteine (PBS, 25°C)	6.3 ± 0.4	3.8 ± 0.1	7.5 ± 0.7
10 mM NAC (PBS, 25°C)	580 ± 9	>1000	>1000
50 mM NAC (PBS, 25°C)	160 ± 2	500 ± 8	>1000
None (PBS, 37°C)	>1000	>1000	>1000
10 mM NAC (PBS, 37°C)	550 ± 30	>1000	>1000
50 mM NAC (PBS, 37°C)	66 ± 5	220 ± 30	530 ± 40
None (CAMHB, 37°C)	>1000	>1000	>1000
10 mM NAC (CAMHB, 37°C)	200 ± 40	670 ± 160	>1000
50 mM NAC (CAMHB, 37°C)	52 ± 6	145 ± 15	225 ± 20

The half-life ($t_{1/2}$) of antibiotics was computed from the pseudo-first order rate constant for carbapenem beta-lactam ring opening, measured by following the time-dependence variation of absorbance at 300 nm (values are the mean of four independent experiments), followed in both PBS buffer and in cation-adjusted Mueller-Hinton Broth (CAMHB) and at 25 or 37 °C. L-cysteine was tested as a comparator in experiments performed at 25°C in PBS.
